# *Enterococcus faecium* GEFA01 alleviates hypercholesterolemia by promoting reverse cholesterol transportation *via* modulating the gut microbiota-SCFA axis

**DOI:** 10.3389/fnut.2022.1020734

**Published:** 2022-11-08

**Authors:** Wenfeng Xu, Kaixiang Zou, Ying Zhan, Yunjie Cai, Zhihong Zhang, Xueying Tao, Liang Qiu, Hua Wei

**Affiliations:** ^1^State Key Laboratory of Food Science and Technology, Nanchang University, Nanchang, China; ^2^Centre for Translational Medicine, Jiangxi University of Chinese Medicine, Nanchang, China

**Keywords:** *Enterococcus faecium*, screening, cholesterol-lowering, physiological performance, microbiota, hypercholesterolemia

## Abstract

This study aimed to identify cholesterol-lowering commensal strains from healthy lean individuals and to evaluate the cholesterol-lowering capacity of *Enterococcus faecium* GEFA01 in mice fed a high-cholesterol and high-fat diet. *E. faecium* GEFA01 was isolated from the feces of a healthy lean individual in a selective basal salt medium supplemented with cholesterol. *E. faecium* GEFA01 exhibited a cholesterol removal rate (CRR) of 46.13% by coprecipitation, assimilation, and degradation of cholesterol. Moreover, *E. faecium* GEFA01 significantly decreased the body weight of mice and the levels of serum total cholesterol (TC), low-density lipoprotein cholesterol (LDL-C), hepatic TC, triglycerides (TG), and LDL-C, and increased serum high-density lipoprotein cholesterol (HDL-C) levels in mice fed a high-cholesterol diet compared with the HCD group. We also observed that *E. faecium* GEFA01 significantly downregulated the gene expression of HMG-CoA reductase (*Hmgcr*), *Srebp-1c, Fxr, Shp*, and *Fgf* 15, upregulated the gene expression of low-density lipoprotein receptor (*Ldlr*), *Abcg5*/*8, Abca1*, cholesterol 7 alpha-hydroxylase (*Cyp7a1*), and *Lxr* in the liver of mice in relative to the HCD group, markedly increased the relative abundance of *Lactobacillus, Akkermansia, Bifidobacterium*, and *Roseburia*, and decreased the abundance of *Helicobacter* in the feces. Collectively, we confirmed that *E. faecium* GEFA01 exhibited cholesterol-lowering effects in mice fed a high-cholesterol diet, which was achieved through assimilation, coprecipitation, and degradation of cholesterol, and through modulation of the gut microbiota short-chain fatty acid (SCFA) axis that promoted reverse cholesterol transport and bile acid excretion. Our study demonstrated that *E. faecium* GEFA01 may be used as a probiotic candidate to lower cholesterol levels in the future.

## Introduction

Hypercholesterolemia, a pathological condition characterized by an exaggerated rise in serum cholesterol, plays a critical role in the development of atherosclerosis and is one of the main risk factors for cardiovascular diseases (CVDs). CVDs are the leading cause of mortality worldwide, especially in developed countries (1). Statins are the first-line drugs for the clinical treatment of hypercholesterolemia; however, there have been some side effects accompanied by elevated serum transaminase levels and some muscle diseases (2, 3). Therefore, there is an urgent need to develop novel therapeutics for the prevention and treatment of hypercholesterolemia.

Probiotics are live microorganisms and may confer health benefits to the host when consumed in adequate amounts (4), including improved intestinal health, enhanced immune response, and cancer prevention. Recently, accumulated evidence has shown that probiotics such as *Lactobacillus* and *Bifidobacterium* exert hypocholesterolemic effects (5–11) *via* enzymatic deconjugation of bile acids (12), the assimilation of cholesterol (13), coprecipitation of cholesterol with deconjugated bile (14), the binding of cholesterol to probiotic cell walls (15), the incorporation of cholesterol into probiotic cell membranes during growth (16), the conversion of cholesterol to coprostanol (17), and the production of short-chain fatty acids (SCFAs) during fermentation in the presence of prebiotics (18). Certain strains of *Enterococcus* belonging to lactic acid bacteria (LAB) provide beneficial effects, such as restoration of microbiota balance of the gastrointestinal tract (GIT) with antibiotic-induced dysbiosis (19), antiviral activity (20), antitumor effect (21, 22), cholesterol-lowering effect (23), and immune regulation (24). However, studies on the cholesterol-lowering effect of *Enterococcus* strains are limited and sporadic, and the mechanisms underlying the cholesterol-lowering effect of *Enterococcus* remain to be elucidated.

In this study, we used a selective medium to isolate *Enterococcus* strains from healthy lean individuals, evaluated their cholesterol-lowering properties, and explored the mechanisms of cholesterol-lowering *in vitro* and *in vivo*, with the aim of providing a theoretical basis for the use of probiotics *Enterococcus* during clinical practice in the prevention and treatment of hypercholesterolemia.

## Materials and methods

### Fecal samples of healthy lean individuals

Six healthy volunteers (male and female, 3 each) between the ages of 21 and 23 were recruited from the Laboratory of Jiangxi-OAI Joint Research Institute of Nanchang University. Volunteers should have no intestinal diseases and did not take antibiotics or fermented lactic acid foods within 2 weeks. A sterile PBS solution containing 10% peptone was used to collect fecal specimens from volunteers in the morning, followed by the isolation and identification of cholesterol-lowering strains immediately.

### Isolation and phylogenetic analysis of cholesterol-lowering strains

Fecal specimens were properly diluted and plated on selective medium plates (g/L) (cholesterol 0.1; oxgall salt 0.2; potassium dihydrogen phosphate 2.5; potassium dihydrogen phosphate 2.5; magnesium sulfate 0.2; ammonium nitrate 3.0; ferrous sulfate 0.1; and dipotassium hydrogen phosphate 2.0; Agar 25.0.), which were incubated under anaerobic conditions (5% CO_2_, 10% H_2_, and 85% N_2_) for 48 h at 37°C. Single colonies were selected and cultured anaerobically in MRS (de Man, Rogosa, and Sharpe agar Beijing Solarbio Science and Technology Co. Ltd. Beijing, China) liquid medium for 24 h at 37°C. Selected isolates were then subjected to 16S recombinant deoxyribonucleic acid (rDNA) sequencing with universal primers 27F and 1492R. Amplicons were sequenced by Sangon Biotech Ltd. (Shanghai, China) and compared to the National Center for Biotechnology Information database using the BLAST algorithm (http://blsat.ncbi.nlm.nih.gov/Blast.cgi) to determine their classification. The fresh bacterial solution cultured for 24 h was stored in 15% skim milk and stored in a refrigerator at −80°C.

### Cholesterol removal assay

*Enterococcus* isolates were inoculated into MRS-Thio-Ox-CHOL medium containing 0.3% bovine bile salt, 0.2% sodium mercaptoacetate, and 0.1 mg/ml cholesterol solution in MRS liquid medium (25) and incubated anaerobically at 37°C for 24 h. Bacterial cell pellets and fermentation supernatant were harvested by centrifugation at 12,000 g and 4°C for 10 min. Bacterial cell pellets were resuspended in MRS-THIO broth containing 0.3% oxgall and 0.2 mg/ml lysozyme and incubated at 37°C for 15 min, and then the suspension was crashed by the supersonic method. The fragmented cell solution was maintained by supplementing MRS-THIO broth with 0.3% oxgall to the original volume and centrifuged at 12,000 g and 4°C for 10 min. Cholesterol concentration in the fermentation supernatant, cell efflux solution, and cell fragment suspension were determined by the colorimetric method (26), taking MRS-THIO broth with 0.3% oxgall as standard.

Cholesterol removal rate (CRR) was calculated by using the equation: CRR = (C–C′)/C, where C and C′ were the concentrations of cholesterol in the medium containing free-bacteria and bacteria, respectively. The cholesterol degradation ratio (CDR) was calculated by using the equation: CDR = [C–(C1 + C2 + C3)]/C, where C was the concentration of initial cholesterol, and C1, C2, and C3 were the concentrations of cholesterol in the fermentation supernatant, cellular efflux solution, and cell fragment suspension, respectively (25).

### Analysis of acid and bile salt tolerance

*Enterococcus* isolates were grown in MRS broth adjusted to pH 2.5 and pH 3.0 or MRS supplemented with 0.15 and 0.3% (w/v) oxgall bile, respectively, and incubated anaerobically at 37°C for 0, 3, and 6 h. Viable bacterial cells were enumerated at 0, 3, and 6 h after challenge with acid or oxgall bile. The isolate incubated in MRS broth at pH 7.0 was taken as control. All tests were performed in triplicate.

### Analysis of antioxidant activity

*Enterococcus* isolates were grown in MRS broth, which contained 0.6 or 1.0 mmol/L H_2_O_2_, and incubated anaerobically at 37°C for 0, 3, and 6 h. Viable bacterial cells were enumerated at 0, 3, and 6 h after hydrogen peroxide challenge. The isolate incubated in MRS broth without H_2_O_2_ was taken as control.

### Analysis of antimicrobial activity against pathogenic bacteria

*Enterococcus* isolates were cultured anaerobically at 37°C for 12 h and centrifuged at 4,000 g for 10 min. The harvested fermentation supernatant was filtered using a 0.22-μm-pore-size filter. After spreading of the selected pathogens such as *Staphylococcus aureus, Salmonella, Bacillus cereus, Listeria monocytogenes, E. coli*, and *Enterococcus sakazakii* on the solid LB plate, sterilized oxford cups were placed on the solidified media in LB plates and 200 μl of fermentation supernatant were, respectively, added into oxford cups. After being placed in a refrigerator at 4°C for 2 h, the plates were then cultured at 37°C for 48 h. The presence of an inhibitory zone around the oxford cups indicated that the fermentation supernatant had an antimicrobial activity.

### Analysis of antibiotic susceptibility

The disk diffusion method was used to evaluate the antibiotic susceptibility of an *Enterococcus* isolate according to the method (27). Briefly, the *Enterococcus* isolate was inoculated into MRS broth and incubated at 37°C overnight. Antibiotic discs were placed onto the surface of solid MRS plates where the *Enterococcus* isolate was evenly streaked. Plates were incubated at 37°C for 24 h. The diameter of inhibition zones around the disks was measured using a digital caliper.

### Analysis of simulated gastric and intestinal fluid tolerance

The survival of an *Enterococcus* isolate in the simulated GIT was investigated as previously described with minor modifications (27, 28). To simulate GIT transit, bacterial cells harvested by centrifugation (7,500 × g, 5 min, 4°C) were resuspended in 10 ml of gastric juice (3 g/L of pepsin, pH 3.0) and incubated for 90 min at 37°C with continuous shaking. Then, bacterial pellets were harvested, resuspended in duodenum juice (1% bile salt, pH 8.0), and incubated anaerobically for 10 min at 37°C. Finally, bacterial pellets were harvested once again, resuspended in intestinal juice (0.3% bile salt and 0.1% pancreatin), and incubated anaerobically for 120 min at 37°C. Viable bacterial cells were enumerated before and after juice challenge.

### Analysis of adherence to caco-2 cells

The adhesion assay of the isolates was detected as previously described (29). Caco-2 cells were cultured in high-glucose Dulbecco's Modified Eagle's Medium (Solarbio, Beijing, China) supplemented with 10% (v/v) fetal bovine serum (Cell Max), 100 U/ml penicillin, and 100 mg/ml streptomycin. Caco-2 cells were seeded in a six-well plate at a concentration of 1 × 10^6^ cells/well. Approximately 1 × 10^9^ CFU/ml of *Enterococcus* isolates resuspended in 2 ml Dulbecco's Modified Eagle Medium (DMEM) without antibiotic were placed into each well. Caco-2 cells were incubated at 37°C for 2 h and gently washed three times with D-HANKS. After digestion with 0.25% trypsin-EDTA solution, 1 ml of DMEM supplemented with 10% bovine serum was added to each well to terminate the activity of trypsin. Serial 10-fold dilutions of cell suspensions were plated on MRS agar to determine the number of adherent bacterial cells. MRS plates were incubated at 37°C for 24 h. The adhesion assay was expressed as a percentage, i.e., the ratio between the number of bacterial cells remaining and the total number of bacterial cells initially added to each well (23).

### Animals and diets

Six-week-old male C57BL/6J mice (20 ± 2 g) wild-type littermates were purchased from Hunan Slake Jingda Laboratory Animal Company (Hunan, China). After acclimatization for 1 week in a controlled environment with constant temperature (22 ± 1°C), mice were randomly divided into four groups (*n* = 10): (1) chow diet (CD) group: mice fed with CD were orally administrated with 200 μl of PBS; (2) HCD group: mice fed a high-cholesterol diet received oral administration of PBS; (3) HCD-atorvastatin group: mice fed a high-cholesterol diet were administered with atorvastatin (20 mg/kg); (4) HCD-GEFA01 group: mice fed with a high-cholesterol diet were orally administered with 200 μl of PBS containing 1 × 10^9^ CFU of *Enterococcus faecium* GEFA01. *E. faecium* GEFA01 was cultured anaerobically in MRS at 37°C for 12 h and centrifuged at 6,000 rpm for 5 min, and supernatant was removed. The precipitate was washed twice with 1 × PBS and resuspended in 1 × PBS with an OD_600_ = 1.0 (10^9^ CFU/ml). The composition of the CD included crude protein ≥ 180 g/kg, crude fat ≥ 40 g/kg, crude fiber ≥ 50 g/kg, crude ash ≤ 80 g/kg, calcium 10 ~ 18 g/kg, phosphorus 6 ~ 12 g/kg, Lys ≥ 8.2 g/kg, and Met + Cys ≥ 5.3 g/kg (30). The composition of the high-cholesterol diet included 50% CD, 16.9% lard, 15% sucrose, 10.8% casein, 1.3% cholesterol, 0.3% BA salt, 2% mouse premix, and 3.7% maltodextrin. The weights of mice were recorded before oral administration throughout the whole assay. Activity and live weight were observed every other day during the experimental period. All mice were orally administered with PBS or *E. faecium* GEFA01 once a day for 8 weeks and then sacrificed after fasting for 8 h. Blood samples were collected for blood lipid analysis, as described below. The liver, kidney, spleen, epididymal fat, and visceral fat were weighed, and their weights were calculated relative to the final body weight (organ index). Fecal pellets, ileum, and contents of cecum were also collected and stored at −20°C until analysis.

### Glucose tolerance test and fasting insulin test

For oral glucose tolerance tests (GTTs), mice were given a single dose of glucose (2 g/kg body weight) by oral gavage after fasting for 12 h and tail blood glucose was measured at 0, 30, 60, 90, and 120 min using a blood glucometer. For fasting insulin test, serum insulin was measured by the ELISA method using the kit (Nanjing Jiancheng Bioengineering Institute, China). The equation: HOMA-IR = Fasting insulin (μU/ml) × Fasting glucose (mmol/L)/22.5 was used to calculate the homeostatic model assessment for insulin resistance (HOMA-IR).

### Analysis of lipid

The liver was mechanically homogenized in 0.9 ml of anhydrous ethanol at 4°C and centrifuged at 4,000 rpm for 15 min at 4°C, and the supernatant was collected. Blood samples were allowed to clot at room temperature and centrifuged at 4,000 g for 15 min at 4°C to collect serum. The levels of hepatic total cholesterol (TC) and triglycerides (TG) or serum TC, TG, high-density lipoprotein cholesterol (HDL-C), and low-density lipoprotein cholesterol (LDL-C) were detected using commercially available kits (Nanjing Jiancheng Bioengineering Institute, Jiangsu, China), according to the manufacturer's instructions.

### Total RNA extraction and real-time quantitative polymerase chain reaction

Total RNA from tissues was extracted using RNAiso Plus reagent (TaKaRa Bio, Otsu, Japan) according to the manufacturer's instructions. Complementary DNA (cDNA) was synthesized with PrimeScript RT reagent kit (TaKaRa Bio, Otsu, Japan). Real-time polymerase chain reaction (PCR) was performed following the SYBR^®^ Premix Ex TaqTM II (TaKaRa Bio, Otsu, Japan). Primer sequences are listed in Table 1. For qPCR amplification, the conditions were as follows: 95°C for 2 min, followed by 40 cycles of denaturation at 95°C for 5 s, annealing at 58°C for 30 s, and extension at 72°C for 5 s. Expression levels of target genes were normalized against those of glyceraldehyde-3-phosphate dehydrogenase (GADPH), and fold changes were calculated using the 2^−ΔΔCt^ method.

**Table 1:** Primer sequence for real-time quantitative PCR (RT-qPCR).

**Gene**	**Forward primer (5^′^–3^′^)**	**Reverse primer (5^′^–3^′^)**
*Srebp-2*	CATCCCTTGGGCCAGAAGTT	TCCTTGGCTGCTGACTTGATC
*Hmgcr*	GCTTGGCCTCCATTGAGAT	CTTTGCTAATGCACTCGCTCT
*Ldlr*	TGACTCAGACGAACAAGGCTG	ATCTAGGCAATCTCGGTCTCC
*Cyp7a1*	AGCAACTAAACAACCTGCCAGTA	GTCCGGATATTCAAGGATGCA
*Fxr*	TGAGAACCCACAGCATTTCG	GCGTGGTGATGGTTGAATGTC
*Shp*	CGATCCTCTTCAACCCAGATG	AGGGCTCCAAGACTTCACACA
*Ppar-α*	AACATCGAGTGTCGAATATGTGG	CCGAATAGTTCGCCGAAAGAA
*Srb1*	TGTACTGCCTAACATCTTGGTCC	ACTGTGCGGTTCATAAAAGCA
*Lxr*	CTCAATGCCTGATGTTTCTCCT	TCCAACCCTATCCCTAAAGCAA
*Abcg5*	TCAATGAGTTTTACGGCCTGAA	GCACATCGGGTGATTTAGCA
*Abcg8*	TGCCCACCTTCCACATGTC	ATGAAGCCGGCAGTAAGGTAGA
*Abca1*	GTTGGTCTCCAGAAGGTATT	TTCAGGATGTCCATGTTGT
*Npc1l1*	TTTCTAGGGGCCCTGACCTC	TTGAAAAGCAGCACACGACG
*Fgf15*	ACGGGCTGATTCGCTACTC	TGTAGCCTAAACAGTCCATTTCCT
*Srebp-1c*	GGAGCCATGGATTGCACATT	GGCCCGGGAAGTCACTGT
*Fas*	CAAGGAGGGCAAGATAGATG	TAAGGTTCTGCGACATTCG
*Scd1*	CTGCCTCTTCGGGATTTTCTACT	GCCCATTCGTACACGTGATTC
*Gapdh*	AGGTCGGTGTGAACGGATTTG	GGGGTCGTTGATGGCAACA

### Hematoxylin and eosin staining and oil red O staining

Liver tissues and white fat tissues were fixed in 4% paraformaldehyde, dehydrated, embedded in paraffin, and cut into 5-μm-thick sections. These sections were deparaffinized in xylene and serially diluted ethanol. Then, liver tissues and adipose tissues were stained with hematoxylin and eosin (H&E). Meanwhile, liver tissues were stained with oil red O as previously described (31).

### SCFA and BA analyses

Short-chain fatty acids of cecum content were measured by gas chromatography–mass spectrometry (GC-MS) using an Agilent 7890-7000D instrument (Agilent Technologies, USA) with slight modifications (32). Briefly, 500 mg of cecal contents were mixed with 2.5 ml of solvent with 1 volume of methanol and 1 volume of 0.2% HCl prior to ultrasonication at 250 W for 5 min, and then the mixture was centrifuged at 8,000 rpm for 10 min. The supernatants were used for GC-MS analysis. Approximately 1 ml of samples or standards were automatically injected at an inlet temperature of 240°C and a split ratio of 50:1. The flow rate of helium was 20 cm/s at a temperature of 175°C. The oven temperature program was as follows: initially it was set to 100°C and held for 5 min, then it was increased to 150°C at a rate of 10°C/min and held at 150°C for 3 min. The ion source temperature was 220°C. SCFAs in the cecum were quantified using standard curves of acetic acid, butyric acid, and propionic acid (33). Total bile acids were measured using commercially available kits (Nanjing Jiancheng Bioengineering Institute, Jiangsu, China).

### 16S rRNA gene amplicon sequencing and bioinformatics analysis

Cryovial was used to collect the cecal contents, which were flash-frozen in liquid nitrogen. QIAamp DNA Stool Mini kit was used to extract genomic DNA of the cecal contents (*n* = 6) for the Illumina 16S DNA sequencing, the primer pair 515F (5′-GTGCCAGCMGCCGCGGTAA-3′) and 806R (5′-GGACTACHVGGGTWTCTAAT-3′) was used to amplify the V4 region of the bacteria 16S rRNA genes, and the Illumina Miseq platform was used to characterize the next generation sequencing by Beijing Genomics Institute (BGI, Shenzhen, China) (34).

Sequencing data were analyzed by the Quantitative Insights into Microbial Ecology (http://qiime.org/) pipeline (35). Briefly, low-quality reads and adapter pollution were eliminated by filtering raw data from each sample while the remaining high-quality paired-end reads were combined into tags using Flash (v 1.2.11; http://ccb.jhu.edu/software/FLASH). The tags were then clustered into operational taxonomic units (OTUs) with 97% sequence similarity using UPARSE (http://www.drive5.com/uparse/). OTU representative sequences were classified using Ribosome Database Project (RDP) Classifier v2.2 (36). Finally, α diversity (including total number of OTUs, Chao1 index, Shannon index, and Simpson index) and partial least squares discriminant analysis (PLS-DA) were used to analyze differences among the groups. The linear discriminant analysis effect size (LEfSe) method (http://huttenhower.sph.harvard.edu/galaxy/) was used to identify differentially abundant taxa between the groups, with a significant α-value of 0.05 and an effect size threshold of 2.

### Statistical analysis

All values were expressed as the mean ± standard deviation (SD) of three independent experiments. Statistical analysis was carried out by GraphPad Prism 7. The data were subjected to a one- or two-way analysis of variance (ANOVA), followed by Tukey's multiple-comparison test. A *p* < 0.05 was considered statistically significant.

## Results

### Screening of cholesterol-lowering isolates

**Figure 1:**
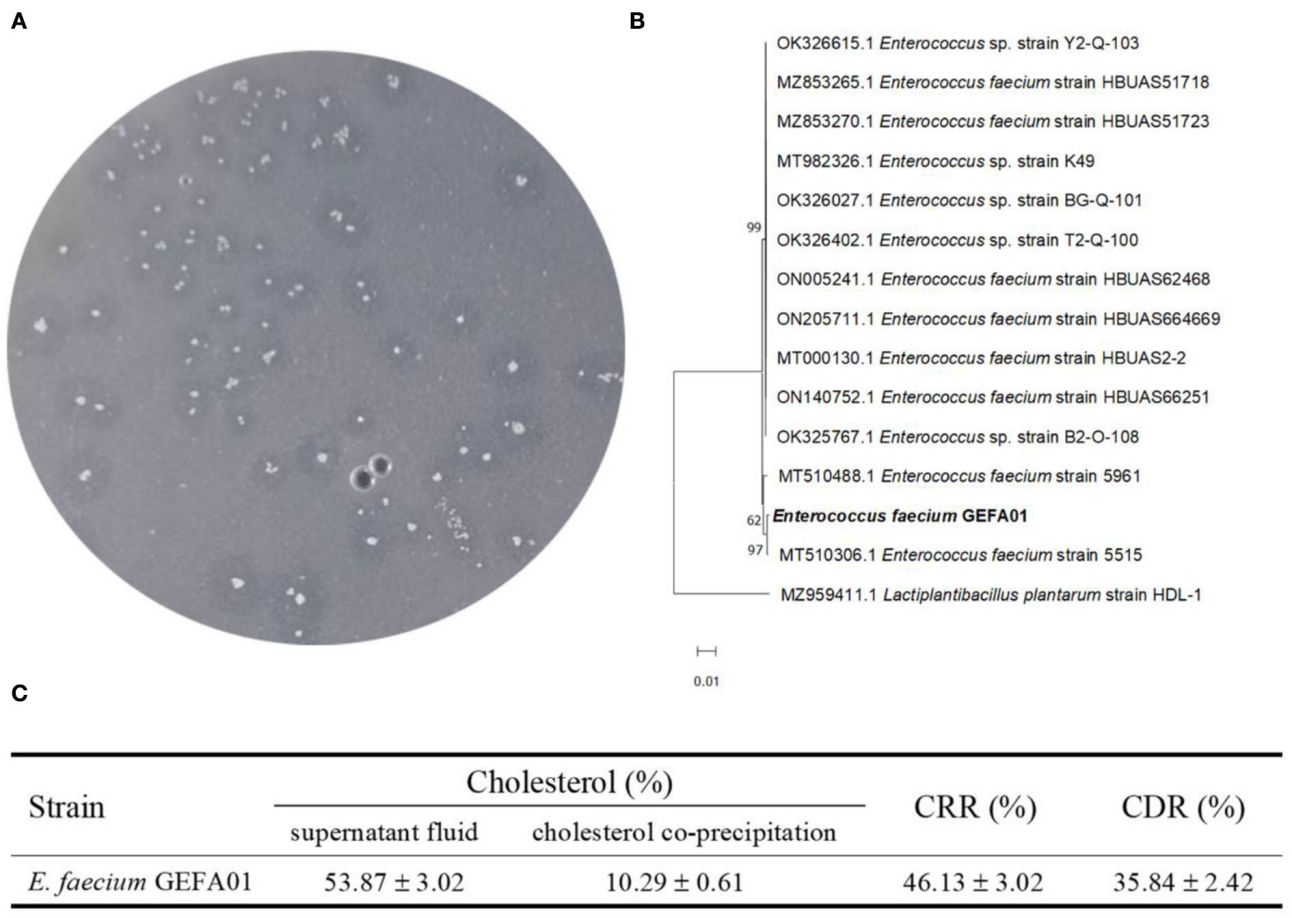
Study on the cholesterol-lowering mechanism of *Enterococcus faecium* GEFA01 *in vitro*. **(A)** Cholesterol-lowering strains were screened and verified by a selecting medium; **(B)** The phylogenetic analysis of *E. faecium* GEFA01; **(C)** The total content of cholesterol (%) in supernatant fluid and cholesterol coprecipitation (including cell-free supernatant and fragmentized cell solution) of GEFA01. Tests were performed in triplicate.

Fecal samples from six healthy Chinese adults were included to isolate cholesterol-lowering strains by using cholesterol and bile as the sole carbon source in the culture medium. A total of three isolates with a transparent zone were screened for the potential utilization of cholesterol. According to near full-length 16S rRNA gene sequencing, phylogenetic analysis showed that one isolate with a transparent zone was identified as *E. faecium* and named strain GEFA01. As shown in Figure 1A, the cholesterol-lowering ability of *E. faecium* GEFA01 was confirmed by the selective medium showing a transparent zone around the bacterial colony. The CRR of *E. faecium* GEFA01 was evaluated by the saponification-colorimetry method, and the results revealed that, in the presence of *E. faecium* GEFA01, 46.13% of cholesterol was reduced in the culture supernatant. In addition, coprecipitated cholesterol (10.29%) was detected, including cholesterol precipitated and resolubilized in the cell efflux solution and cholesterol incorporated into the cell membrane or cell well. Therefore, the CRR and CDR are 46.13 and 35.84, respectively (Figure 1B).

### Evaluation of the physiological properties of *E. faecium* GEFA01 *in vitro*

**Figure 2:**
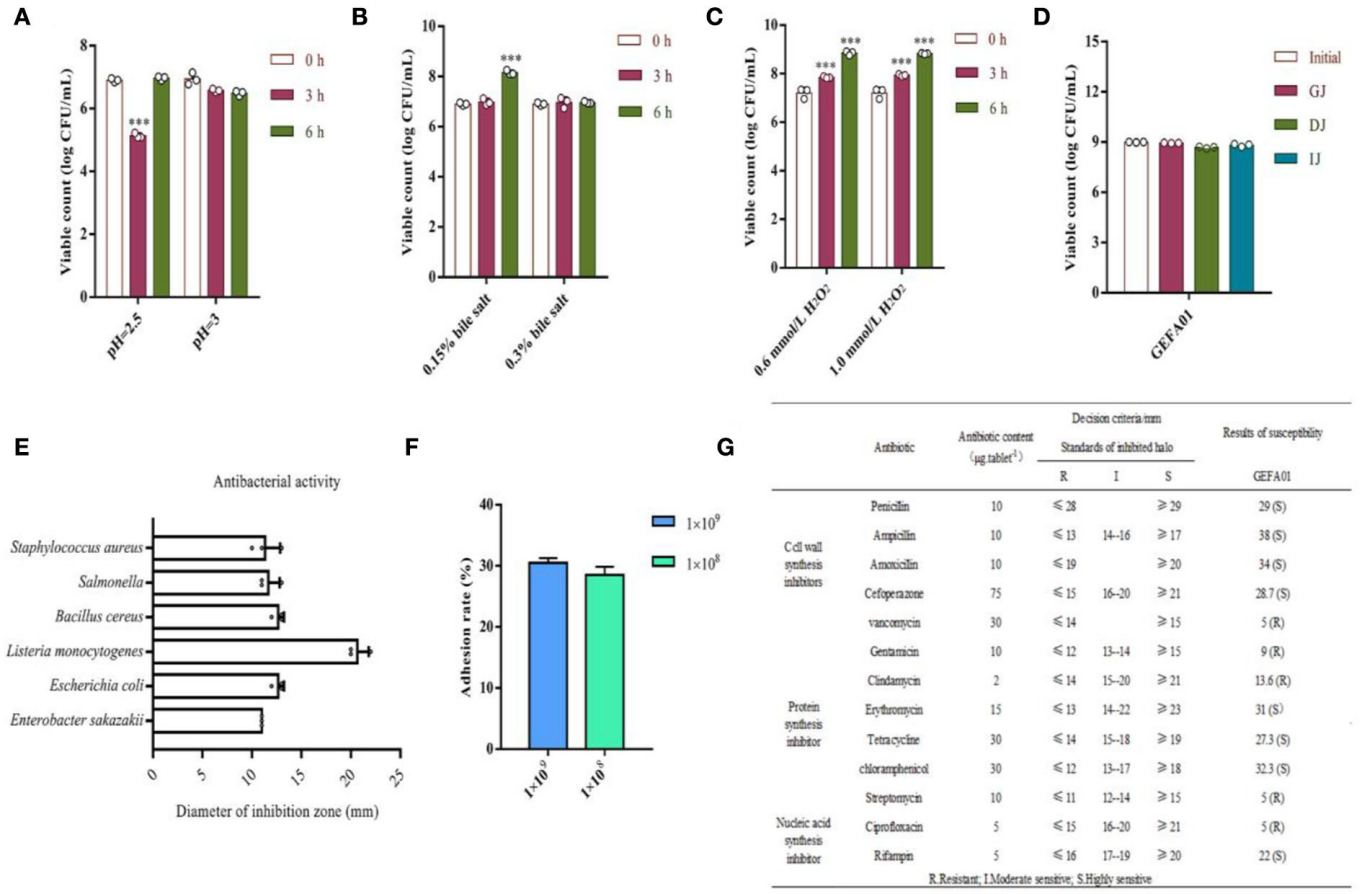
Evaluation of the physiological properties of *E. faecium* GEFA01 *in vitro*. **(A)** Acid resistant; **(B)** bile salt tolerant; **(C)** H_2_O_2_ resistant; **(D)** tolerance of simulated gastroenteric fluid; **(E)** antagonizing pathogenic bacteria; **(F)** adhesion rate to Caco-2 cells of different bacterial quantity; and **(G)** susceptibility tests to 13 antibiotics. Data were expressed as mean ± standard error of the mean (SEM). *p < 0.05, **p < 0.01, ***p < 0.001. Tests were performed in triplicate.

To verify the safety of GEFA01, the physiological properties of *E. faecium* GEFA01 were investigated. As shown in Figure 2, for acid resistance, the viable counts of GEFA01 treated at pH 2.5 for 3 h decreased significantly compared with non-treated cells, no significant difference was observed between the viable counts of GEFA01 treatment at pH 2.5 for 0 and 6 h, and there was no significant difference among viable counts of GEFA01 treated at pH 3.0 for 0, 3, and 6 h. For bile salt resistance, GEFA01 was tolerant to 0.15 and 0.3% bile salts, and the viable counts of GEFA01 treated with 0.15% bile salt for 6 h were significantly increased compared with 0 h. For antioxidant activity, the viable counts of GEFA01 treated with 0.6 or 1.0 mmol/L H_2_O_2_ for 3 and 6 h, respectively, were significantly increased in relative to the viable counts of GEFA01 at 0 h. For simulated gastroenteric fluid tolerance, no significant differences in the viable counts of GEFA01 were observed in simulated gastroenteric fluid compared with the viable counts of GEFA01 at 0 h. For antimicrobial activity, GEFA01 was grown on a plate containing *S. aureus, Salmonella, B. cereus, L. monocytogenes, E. coli*, and *E. sakazakii*, all inhibition zone diameters on the plate were larger than 1 cm, and the largest diameter of inhibition zone was 2.1 cm on the plate of *L. monocytogenes*. For adhesion activity, there was no statistically significant difference in the adhesion of GEFA01 (1 × 10^8^ vs. 1 × 10^9^ CFU) to Caco-2 cells, which showed adhesion percentages of up to ~30%. For antibiotic susceptibility, GEFA01 was sensitive to the majority of the 13 kinds of broad-spectrum antibiotics including penicillin, ampicillin, amoxicillin, cefoperazone, vancomycin, gentamicin, clindamycin, erythromycin, tetracycline, chloramphenicol, streptomycin, ciprofloxacin and rifampin, except for vancomycin, gentamicin, clindamycin, streptomycin, and ciprofloxacin.

### *E. faecium* GEFA01 reduced cholesterol levels in hypercholesterolemic mice

**Figure 3:**
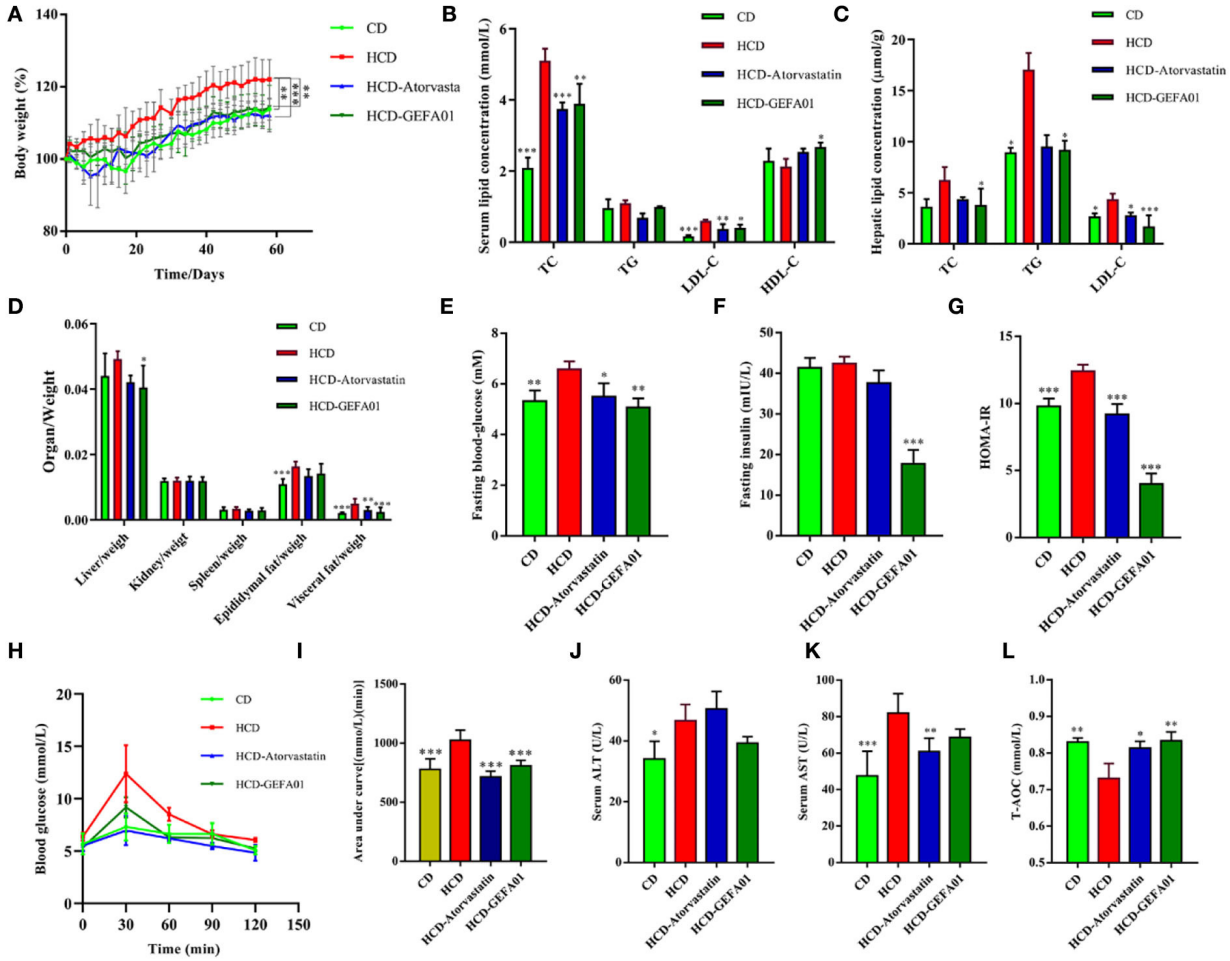
*E. faecium* GEFA01 reduced the cholesterol level in hypercholesterolemic mice. **(A)** Body weight gain curves; **(B)** serum lipid levels including total cholesterol (TC), triglycerides (TG), low-density lipoprotein cholesterol (LDL-C), and high-density lipoprotein cholesterol (HDL-C); **(C)** hepatic lipid levels; **(D)** organ/weight; **(E)** blood glucose level after overnight fasting at 8 weeks; **(F)** fasting insulin level in plasma by ELISA; **(G)** homeostatic model assessment-insulin resistance (HOMA-IR) index, calculated by fasting blood glucose (mmol/L) × fasting insulin (mU/L)/22.5; **(H)** glucose tolerance test (GTT); **(I)** area under glucose tolerance curve; **(J)** serum alanine aminotransferase (ALT) and **(K)** aspartate aminotransferase (AST) levels; and **(L)** serum T-AOC, total antioxidant capacity. Data were expressed as mean ± SEM: *n* = 4 animals per group; *p < 0.05, **p < 0.01, ***p < 0.001, all groups were compared to the HCD group.

To investigate whether strain GEFA01 reduced the serum levels of cholesterol in mice, mice fed a high-cholesterol and high-fat diet were orally administrated with strain GEFA01 for 8 weeks. As shown in Figure 3, compared to mice in the CD group, mice in the HCD group exhibited a significant increase in weight, serum TC and LDL-C levels, and hepatic TG and LDL-C levels. When fed with GEFA01, mice in the HCD-GEFA01 group showed a significant decrease in weight, serum TC and LDL-C levels, and hepatic TC, TG, and LDL-C levels, and showed a significant increase in serum HDL-C levels. HCD-treated mice also exhibited significant increases in both fasting glucose and insulin levels compared to CD-treated mice, when treated with GEFA01, decreased fasting glucose and insulin levels were observed in mice of the HCD-GEFA01 group, which were consistent with the HOMA-IR index in mice from the HCD-GEFA01 group.

### *E. faecium* GEFA01 alleviated liver damage and steatosis in hypercholesterolemic mice

**Figure 4:**
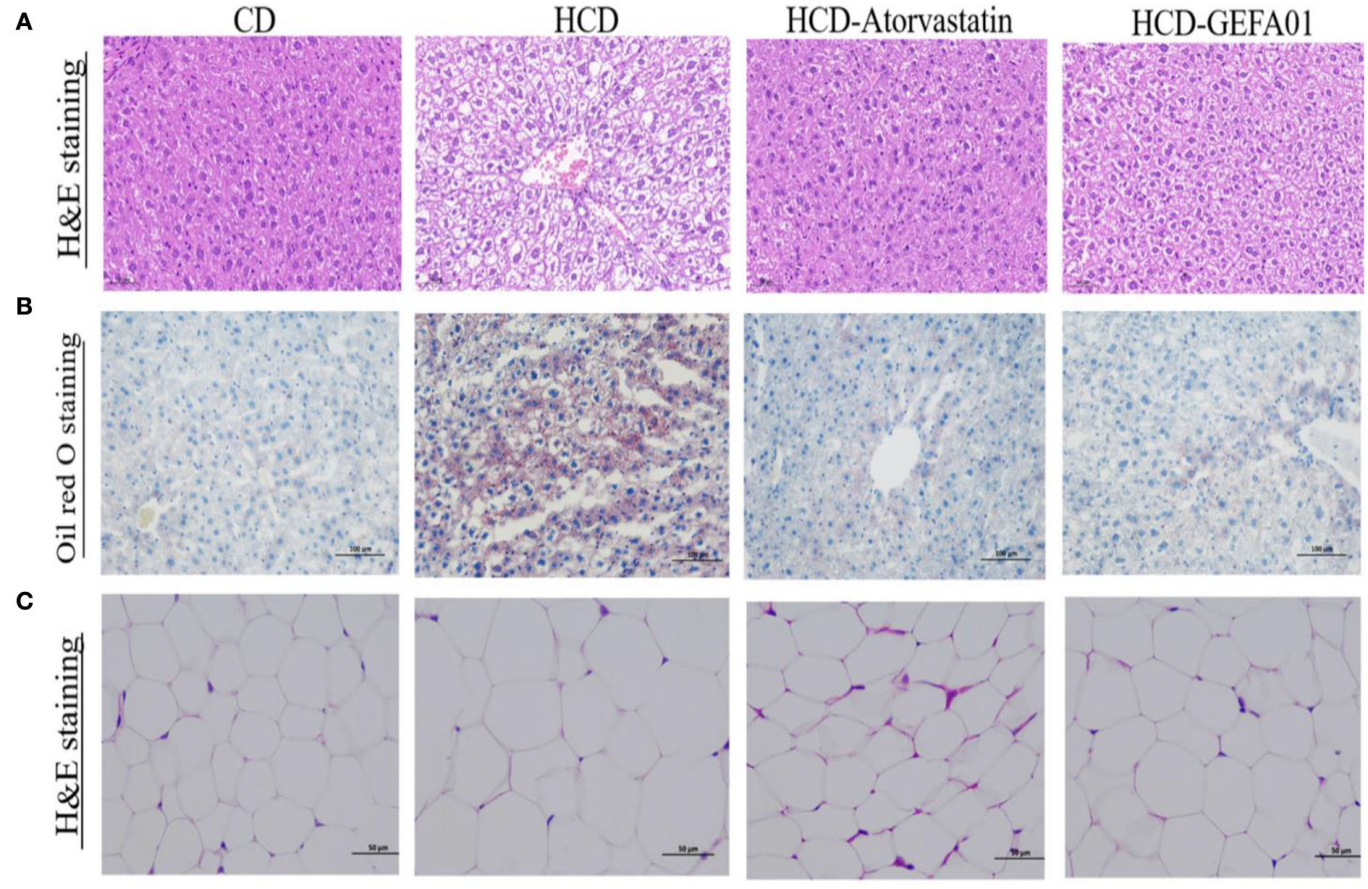
*E. faecium* GEFA01 alleviated liver damage and steatosis in hypercholesterolemic mice. **(A)** Morphology analysis of liver tissues by hematoxylin and eosin (H&E) staining; **(B)** morphology analysis of liver tissues by Oil red O staining; and **(C)** morphology analysis of adipose tissues by H&E staining. *n* = 3 animals per group. A and C images represent the sections observed under 20× magnification whereas B images are observed under 10× magnification. Scale bars of 50 and 100 μm are allotted for 20× and 10× magnifications, respectively.

To evaluate whether *E. faecium* GEFA01 alleviated liver damage and steatosis caused by a high-cholesterol and high-fat diet, histopathological changes of the liver were detected by H&E and oil red O staining. As indicated in Figure 4, hepatocyte ballooning and hepatic vacuoles were observed in HCD-fed mice from the morphological analysis of liver tissues by H&E staining, as expected; however, in HCD-GEFA01-fed mice, the hepatocytes had markedly decreased abnormal ballooning. As demonstrated by oil red O staining, lipid droplets in the liver of HCD-fed mice also showed high contents of lipid. In contrast, lipid deposition in HCD-GEFA01-fed mice was significantly lower. In addition, a remarkable increase in adipocyte size was observed in the liver tissues of the HCD group compared to the CD group, while a remarkable reduction of lipid droplets was observed in the HCD-GEFA01 group.

Consistent with histological changes, serum aspartate aminotransferase (AST), and alanine aminotransferase (ALT) levels of mice in the HCD group were significantly higher than those of mice in the CD group, and after treatment with GEFA01, mice in the HCD-GEFA01 group exhibited a significant decrease in the levels of AST and ALT as compared with the HCD group.

### *E. faecium* GEFA01 regulated gene expression associated with lipid metabolism in hypercholesterolemic mice

**Figure 5:**
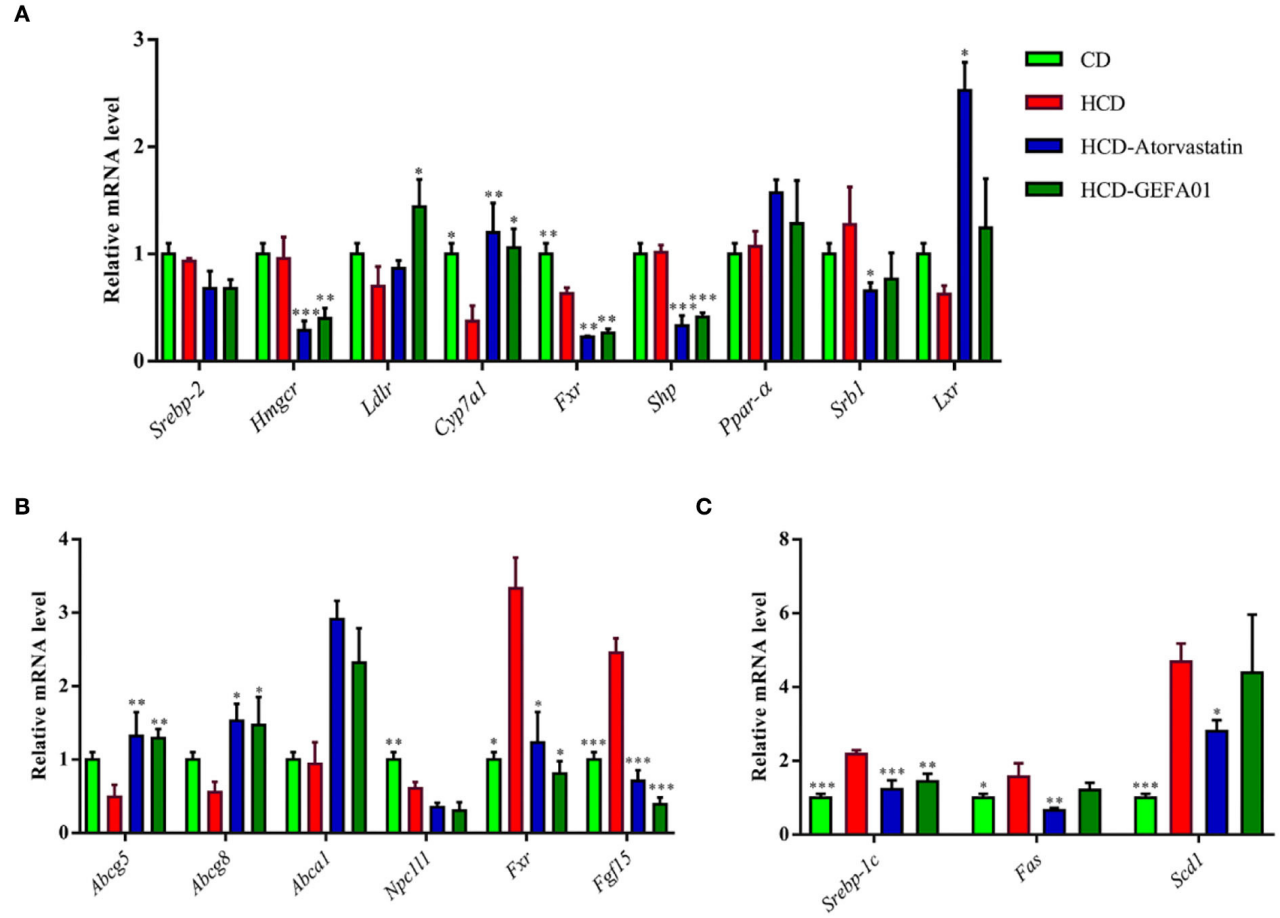
*E. faecium* GEFA01 regulated the expression of genes associated with lipid metabolism in hypercholesterolemic mice. **(A)** The effects of *E. faecium* GEFA01 on the expression of key genes involved in bile acid and cholesterol metabolism of the liver; **(B)** the effects of *E. faecium* GEFA01 on the expression of key genes about cholesterol absorption and efflux metabolism of the ileum; and **(C)** the expression of regulators related to lipid synthesis at messenger ribonucleic acid (mRNA) levels using RT-qPCR. Data were expressed as mean ± SEM: *n* = 3 animals per group; *p < 0.05, **p < 0.01, ***p < 0.001, all groups were compared to the HCD group.

To investigate how *E. faecium* GEFA01 reduced the serum levels of cholesterol in mice, expression profiles of cholesterol metabolism and transport-associated genes were detected by real-time quantitative PCR (RT-qPCR). The results demonstrated that a significant decrease in the expression level of hepatic cholesterol 7 alpha-hydroxylase (*Cyp7a1*), and there was no obvious difference in the expression of hepatic HMG-CoA reductase (*Hmgcr*), scavenger receptor-BI (*Sr-bI*), and the low-density lipoprotein receptor (*Ldlr*) in the HCD group compared with the CD group (Figure 5A). However, the expression level of *Cyp7a1* and *Ldlr* was significantly increased, and the expression level of *Hmgcr* was inhibited by GEFA01 relative to the HCD group (Figure 5A). In addition, no significant differences in hepatic *Srebp2* and *Lxr* expression were observed among the CD, HCD, and GEFA01 groups, and the expression level of both hepatic *Fxr* and *Shp* was inhibited by GEFA01 compared with the HCD group (Figure 5A). At the same time, after oral administration with *E. faecium* GEFA01, a significant increase in the expression level of ileum *Abcg5*/*8* and *Abca1* while a significant decrease in the expression level of ileum *Fxr* and *Fgf15* was observed compared with the HCD group (Figure 5B). In addition, the expression of regulators related to lipid synthesis such as *Srebp-1c, Fas*, and *Scd1* was significantly decreased after oral administration with GEFA01 compared with the HCD group (Figure 5C).

### *E. faecium* GEFA01 increased fecal acetate and bile acid levels in hypercholesterolemic mice

**Figure 6:**
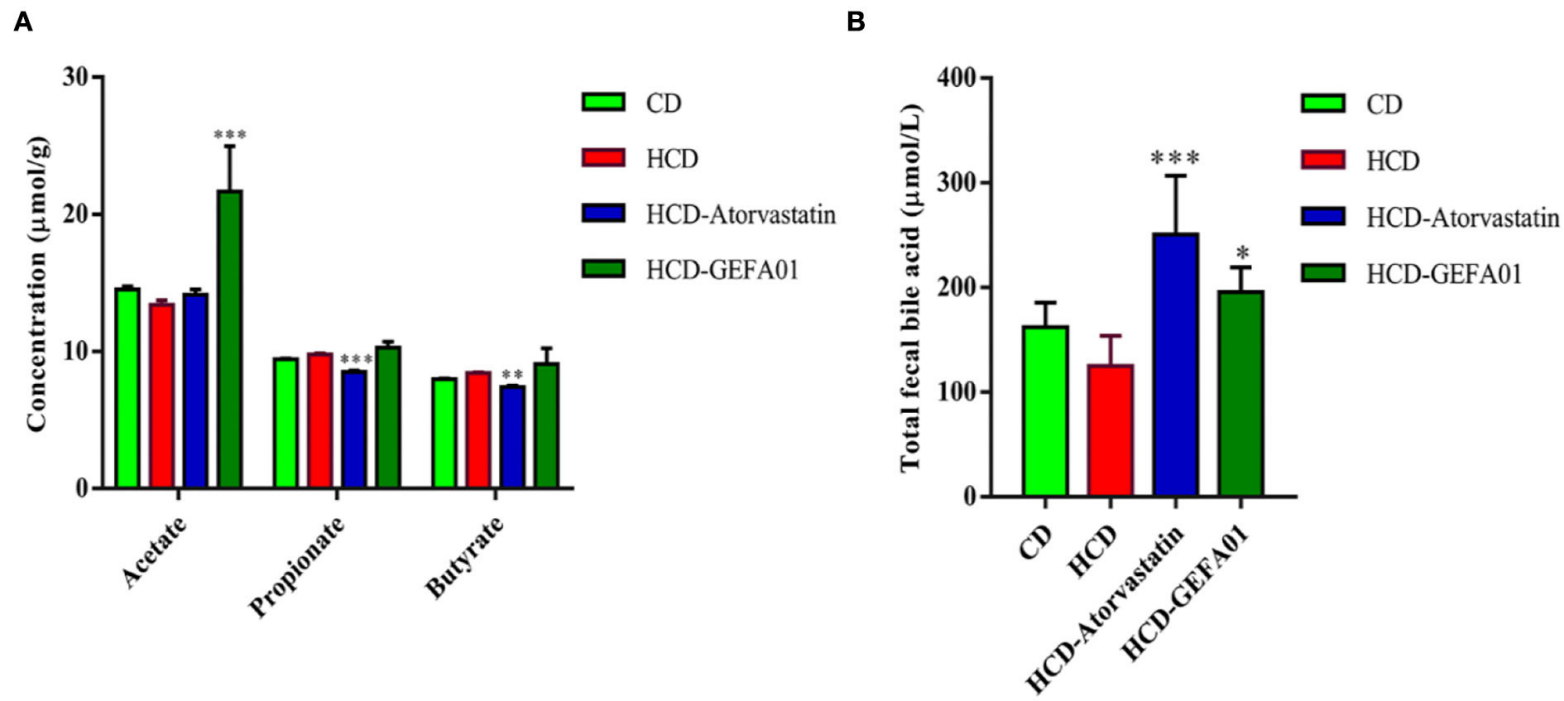
*E. faecium* GEFA01 increased fecal acetate and bile acid level of hypercholesterolemic mice. **(A)** Short-chain fatty acid (SCFA) contents in feces and **(B)** total bile acid contents in feces. Data were expressed as mean ± SEM: *n* = 4 animals per group; *p < 0.05, **p < 0.01, ***p < 0.001, all groups were compared to the HCD group.

Previous studies have shown that SCFAs are capable of decreasing plasma cholesterol (37). As shown in Figure 6A, the contents of fecal acetate in hypercholesterolemic mice after oral administration with GEFA01 were significantly increased compared to those in the HCD group, while the concentration of propionate and butyrate levels was not significantly different between the HCD and HCD-GEFA01 groups. Additionally, the contents of total bile acid in the feces of hypercholesterolemic mice after oral administration with GEFA01 were also significantly increased compared to those in the HCD group.

### *E. faecium* GEFA01 modulated gut microbiota in hypercholesterolemic mice

**Figure 7:**
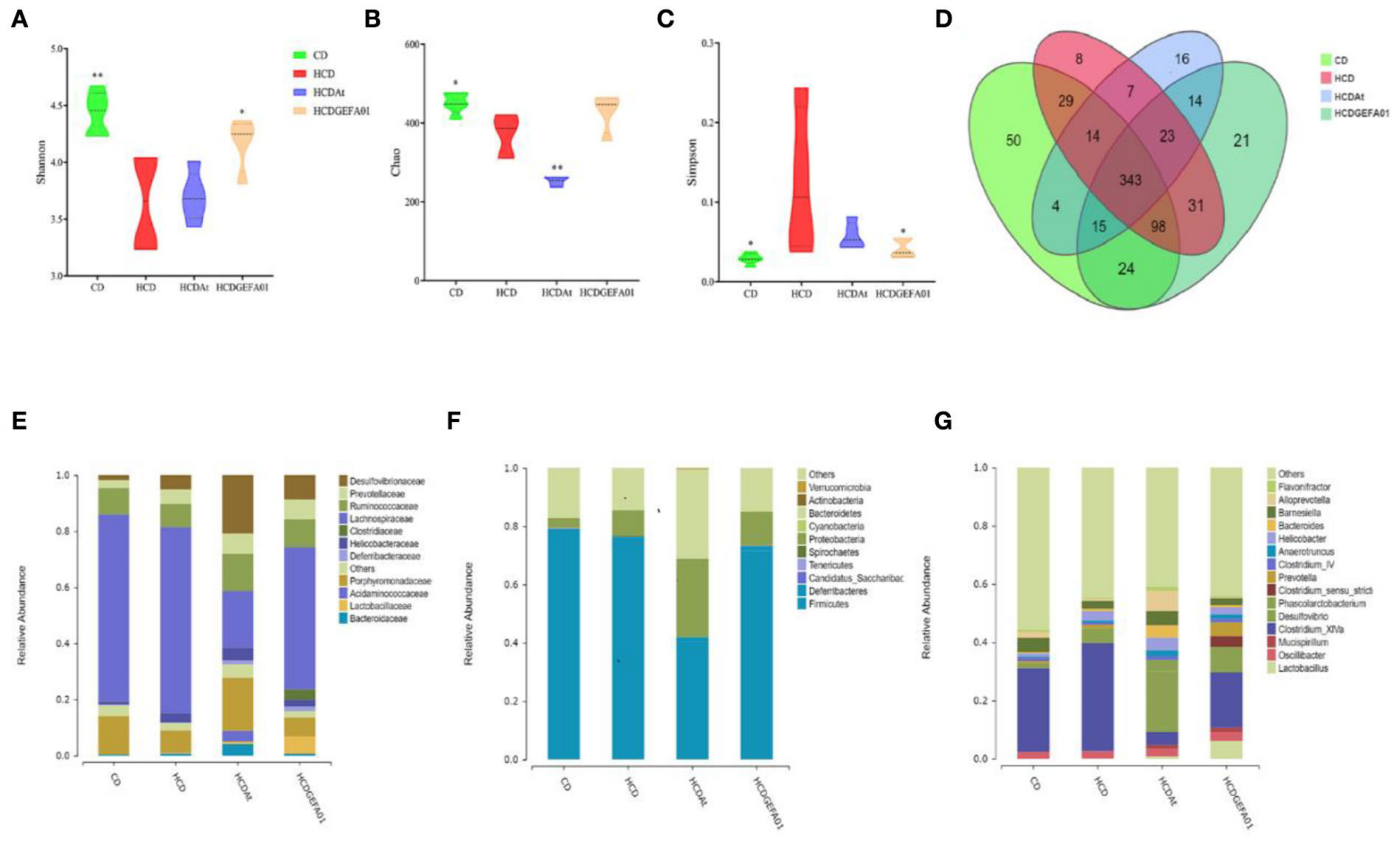
*E. faecium* GEFA01 modulated gut microbiota in hypercholesterolemic mice. **(A)** The microbiota diversity was measured by the Shannon index; **(B)** the microbiota richness was measured by the Chao index; **(C)** the microbiota diversity was measured by the Simpson index; **(D)** Venn diagram representation of the number of operational taxonomic units (OTUs) at the genus level from the chow diet (CD), HCD, HCDAt, and HCDGEFA01 groups; **(E)** relative abundance at the family level; **(F)** relative abundance at the phylum level; and **(G)** relative abundance at the genus level. Data were expressed as mean ± SEM: *n* = 6 animals per group; *p < 0.05, **p < 0.01, all groups were compared to the HCD group.

**Figure 8:**
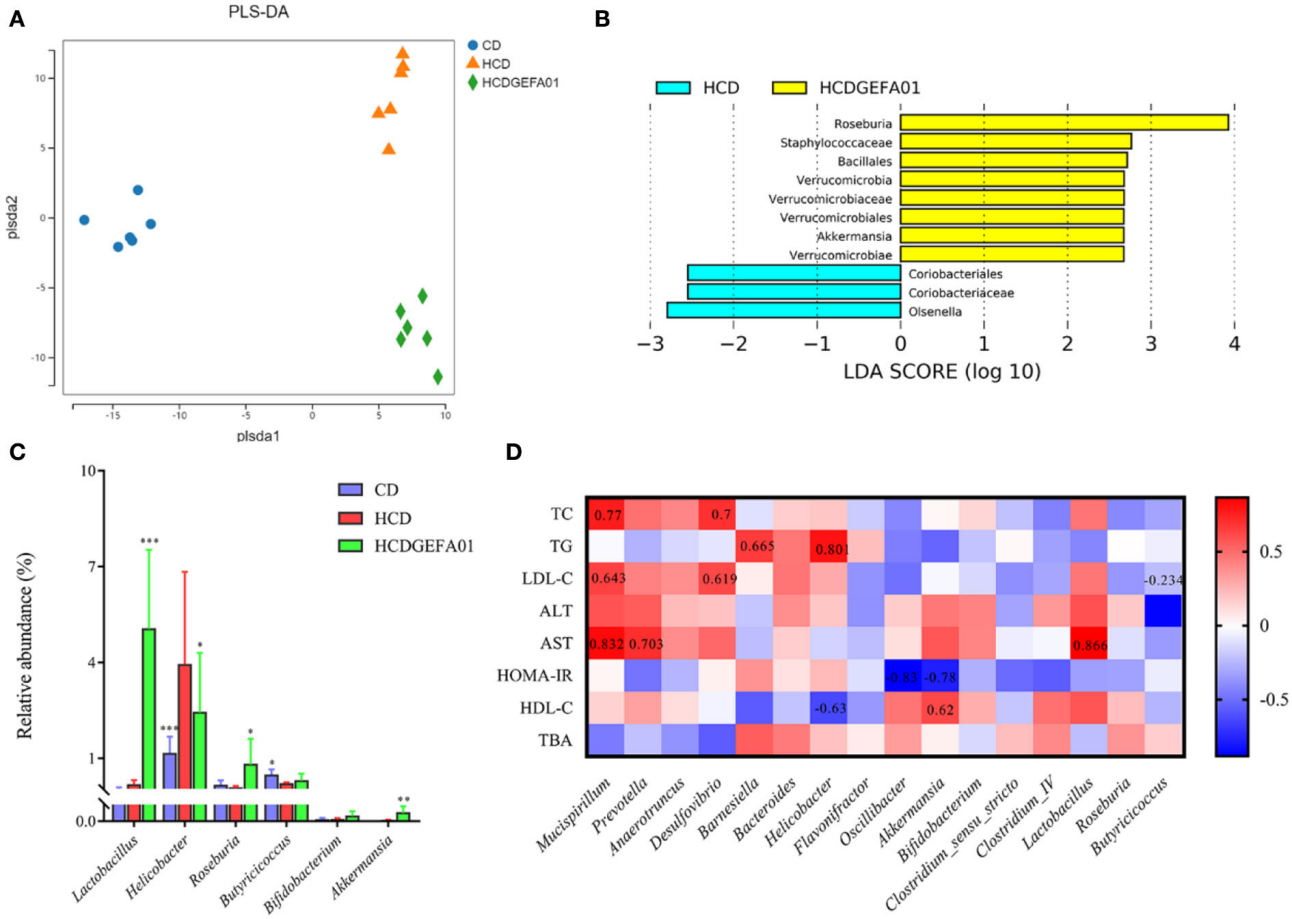
A correlation between perturbed gut bacteria genera and metabolic disorder-related indices. **(A)** partial least squares discriminant analysis (PLS-DA) plot of gut microbiota; **(B)** linear discriminant analysis effect size (LEfSe) analyzed the most differential taxons between the HCD and HCDGEFA01 groups; **(C)** Relative abundance at the genus level, including *Lactobacillus, Helicobacter, Roseburia, Butyricicoccus, Bifidobacterium*, and *Akkermansia*. **(D)** Spearman's correlation analysis was calculated between 16 identified bacteria genera and cholesterol metabolism traits. Data were expressed as mean ± SEM: *n* = 6 animals per group; *p < 0.05, **p < 0.01, ***p < 0.001, all groups were compared to the HCD group. The red and blue blocks represent positive and negative correlations, respectively.

It is reported that lipid metabolism could be affected by the gut microbiota (38). Thus, the influence of *E. faecium* GEFA01 on gut microbiota composition was explored by 16S rRNA gene amplicon sequencing. These results suggested that lower bacterial diversity (as measured by the Shannon and Simpson indexes) and decreased bacterial richness (as measured by the Chao index) were observed in HCD mice at 8 weeks compared to those in the CD mice. After treatment with GEFA01, higher bacterial diversity and increased bacterial richness were observed (Figures 7A–C). Meanwhile, a Venn diagram (Figure 7D) was used to better characterize the shared richness among the four groups. Compared with the HCD group, a significant increase abundance of *Lactobacillaceae* at family level were detected in HCD-GEFA01 mice (Figure 7E). Moreover, data analysis was expanded to the 15 dominant genus of intestine microbial community structure. As shown in Figure 7F, compared with the CD group, a lower relative abundance of Bacteroidetes and no significant difference of Firmicutes were detected in HCD mice. Supplementation with atorvastatin and GEFA01 reversed this situation. Intriguingly, both the HCD-At and HCD-GEFA01 groups had enriched amounts of *Verrucomicrobia* compared with HCD mice. *E. faecium* GEFA01 ameliorated microbiota dysbiosis by increasing the relative abundance of *Lactobacillus, Roseburia, Butyricicoccus, Bifidobacterium*, and *Akkermansia* at the genus level compared with HCD mice (Figures 7G, 8C).

### Correlation between perturbed gut bacteria genera and metabolic disorder-related indices

To further evaluate the effect of GEFA01 on the composition of gut microbiota among the groups, bacterial structures were analyzed using PLS-DA, Spearman's correlation analysis, and LEfSe analysis. PLS-DA analyses showed a significant clustering of the microbiota among the CD, HCD, and HCD-GEFA01 groups. The microbiota composition was markedly distinct from the HCD group after *E. faecium* GEFA01 treatment, indicating that *E. faecium* GEFA01 can effectively shape the gut microbiota community (Figure 8A). Moreover, LEfSe analysis revealed that GEFA01 decreased the abundance of *Coriobacteriales* and *Olsenella* and markedly increased the abundance of *Roseburia, Verrucomicrobia*, and *Akkermansia* compared with the HCD group (Figure 8B). At the genus level, GEFA01 treatment conspicuously increased the abundance of *Lactobacillus, Roseburia, Butyricicoccus, Bifidobacterium*, and *Akkermansia*, which positively and negatively correlate with SCFAs and obesity, respectively, while simultaneously decreasing the abundance of *Helicobacter* (Figure 8C).

Spearman's correlation analysis was used to clarify the relationship of physiological index and bacterial abundance. As indicated in Figure 8D, at the genus level, the abundance of *Mucispirillum, Prevotella, Anaerotruncus, Desulfovibrio, Barnesiella*, and *Helicobacter* was positively correlated with the levels of serum TC, TG, LDL-C, ALT, AST, and HOMA-IR, while their abundance was negatively correlated with the levels of serum HDL-C and fecal TBA. The abundance of *Oscillibacter, Akkermansia, Bifidobacterium, Clostridium_IV, Lactobacillus, Roseburia*, and *Butyricicoccus* presented a negative correlation with the levels of serum TC, TG, LDL-C, ALT, and HOMA-IR, and a positive correlation with the levels of serum HDL-C and fecal TBA, suggesting that *E. faecium* GEFA01 treatment improved cholesterol metabolism, liver damage, HOMA-IR, and bile acid metabolism in hypercholesterolemic mice by altering the gut microbiota.

## Discussion

Probiotics, such as *Lactobacillus* and *Bifidobacterium*, provide health benefits including cholesterol-lowering effects for the host (7), but a few *Enterococcus* strains were reported. In the present study, *E. faecium* GEFA01 was isolated from healthy lean individuals on the selective media. Desirable physiological performance of *E. faecium* GEFA01 was confirmed by the properties of acid resistance, bile salt tolerance, antibiotic susceptibility, antimicrobial activity, antioxidant ability, and its adherence to Caco-2 cells. Moreover, *E. faecium* GEFA01 alleviated hypercholesterolemia by ameliorating gut microbiota dysbiosis and regulating expression levels of cholesterol metabolism and transport-associated genes in mice fed a high-cholesterol diet.

Various mechanisms have been proposed for cholesterol removal using probiotics, such as deconjugation of bile salts by bile salt hydrolase (BSH) activity and assimilation of cholesterol. Probiotic lactic acids, such as *Lactobacillus* and *Enterococcus*, possess BSH activity (39). The BSH enzyme catalyzes bile salt deconjugation by liberating the glycine and/or taurine moiety from the side chain of the steroid core and generating deconjugated bile salts (40). *Lactobacillus* spp. and *Enterococcus* removed cholesterol from media *in vitro* by coprecipitation of deconjugated bile salts with cholesterol (41). In addition, probiotics also remove cholesterol by incorporating cholesterol into the cell membranes during growth, cholesterol entering the cell membrane increases the concentration of saturated and unsaturated fatty acids, resulting in an increase in membrane strength, followed by higher cell resistance (16). Probiotics also exert a hypocholesterolemic effect by assimilating cholesterol during growth, which reduces the amount of cholesterol available for intestinal absorption (17). In this study, 53.87% of cholesterol remained in the culture media, indicating that 46.13% of cholesterol was removed by GEFA01. Further study showed that only 10.29% of cholesterol was detected in a supernatant released from the damaged cell. Thus, we inferred that this part of cholesterol was originated from cholesterol coprecipitated with deconjugated bile salts and incorporated into cell membranes. Meanwhile, the remaining 35.84% of cholesterol undetected may be converted into other substances by GEFA01. Therefore, we proposed that the mechanisms for cholesterol removal should include assimilation, coprecipitation, and degradation of cholesterol by *E. faecium* GEFA01.

It is suggested that a potential probiotic with beneficial effects should exhibit some desirable properties (42), such as acid and bile tolerance, adhesion to mucosal and epithelial surfaces, antibacterial activity against pathogenic bacteria, and antibiotic resistance. It is considered that probiotics should be able to tolerate low pH (2–3) for 2 h and high bile salts (0.3%, wt/vol) for 12 h according to Usman and Hosono (43). In the present study, *E. faecium* GEFA01 maintained its viability at pH 3.0 for more than 2 h, and its viability was significantly decreased at pH 2.5 for 3 h and then increased for 6 h, indicating that *E. faecium* GEFA01 needed to adapt to pH lower than 2.5 before it grows. Bile salts are potent antimicrobial agents and are the most serious obstacles for probiotics in the small intestine. Here, *E. faecium* GEFA01 retained its viability in MRS broth supplemented with 0.15 and 0.3% oxgall bile, respectively, and grew in MRS supplemented with 0.15% for 6 h. Furthermore, after exposure to simulated gastrointestinal transit (i.e., GJ, DJ, and IJ), *E. faecium* GEFA01 was able to survive exposure of protease, bile salt, and acid for 2 h. The antimicrobial activities of probiotic strains are essential to prevent the infection or invasion of pathogenic bacteria (44). *E. faecium* GEFA01 possessed antimicrobial effects on *S. aureus, Salmonella, B. cereus, L. monocytogenes, Escherichia coli*, and *E. sakazakii*, and showed the strongest antimicrobial activity against *L. monocytogenes*, which indicated that *E. faecium* GEFA01 might play a certain competitive inhibitory effect on pathogenic bacteria and colonize well in the human intestinal tract. The ability of microorganism to adhere to host cells is essential to permanently survive in the host's intestine (45). The human epithelial cell line Caco-2 possesses morphologic and physiologic characteristics of normal human enterocytes and has been widely used to assess adherence ability of probiotics (46–48). The adhesion rate of *E. faecium* GEFA01 (1 × 10^8^ or 1 × 10^9^ CFU/ml) to Caco-2 cells was high up to ~30%, which is larger than most of probiotics (23, 49). It is necessary to evaluate the safety of natural isolates from healthy lean individuals due to the importance of *Enterococci* in the dairy industry, particularly in variety products of cheese (50). Resistance against different antibiotics is considered as one of the most important factors for evaluating the safety of *Enterococcus* strains (51). *E. faecium* GEFA01 showed sensitivity to 13 kinds of broad-spectrum antibiotics, except for some antibiotics including cell wall synthetic inhibitors (vancomycin), the protein synthetic inhibitors (gentamicin, clindamycin, and streptomycin), and the nucleic acid synthetic inhibitors (ciprofloxacin), which indicates that *E. faecium* GEFA01 may be used safely in food and dairy products.

Accumulating evidence has demonstrated that high-cholesterol and high-fat diet induces serum lipid abnormalities, impaired glucose and insulin tolerance, and intracellular lipid accumulation. Probiotic therapy improves high-cholesterol diet-induced-lipid abnormalities, steatohepatitis, and insulin resistance in mice (52). In our previous study, *E. faecium* WEFA23 from infants improves high-fat diet-induced metabolic syndrome in mice (53). Consistent with a previous study (23, 54, 55), *E. faecium* GEFA01 significantly decreased serum and hepatic TC and LDL-C levels and increased serum HDL-C level in mice fed a high-cholesterol diet for 8 weeks. Additionally, *E. faecium* GEFA01 effectively reduced lipid deposition, glucose intolerance, and liver damage, indicating that *E. faecium* GEFA01 could effectively alleviate high-cholesterol diet-induced metabolic syndrome in mice.

The concentration of cholesterol circulating in the blood is regulated by the balance between exogenous uptake and endogenous synthesis. Exogenous cholesterol is taken up *via* the Niemann-Pick C1-like 1 (NPC1L1) protein located on the apical surface of enterocytes and then taken up by LDL receptors (LDLR) located on the basal surface of polarized cells (56). While endogenous cholesterol synthesis is mainly regulated by 3-hydroxy-3-methyglutaryl coenzyme A reductase (HMGCR) in the liver, which is the rate-limiting enzyme in *de novo* cholesterol synthesis (57). Cholesterol-related genes, such as *Hmgcr* and *Ldlr*, are mainly regulated by sterol-regulatory element-binding protein 2 (SREBP-2) (58). In the liver, cholesterol is converted to bile acids by the rate-limiting enzyme cholesterol 7α-hydroxylase (CYP7A1). Bile acids activate the nuclear farnesoid X receptor (FXR), and induce the expression of a small heterodimer partner (SHP), which represses the expression of CYP7A1 (59). The liver X receptor (LXR) plays an important role in reversing cholesterol transport and reducing cholesterol uptake (60). Excess cholesterol in the liver is excreted into the intestine or bile by the ABC subfamily G member 5 and member 8 (ABCG5/8) transporters (57). Cholesterol can also be effluxed from the liver or macrophages through ABC subfamily A member 1 (ABCA1) and subfamily G member 1 (ABCG1) to form mature HDL (61). Previous studies demonstrated that *E. faecium* Strain 132 and *Lactobacillus paracasei* Strain 201 upregulated the expression of *Cyp8b1* and *Cyp7a1* genes, respectively, which reduced cholesterol levels in rat (62). Our previous study also showed that *E. faecium* WEFA23 improved hyperlipidemia *via* modulating genes relevant to the decomposition of cholesterol (*Cyp7a1*), synthesis of cholesterol (*HMGCoAS, Scd1*), and transportation (*Ldlr* and *Srb1*) of cholesterol in rats fed a high-fat diet (63). In this study, treatment with atorvastatin and GEFA01 significantly downregulated the expression of *Hmgcr, Npc1l1, Fxr*, and *Shp* and upregulated the expression of *Ldlr, Abcg5*/*8, Abca1, Cyp7a1*, and *Lxr* compared to those in the HCD group, indicating that *E. faecium* GEFA01 induced the flux of peripheral cholesterol to the liver, downregulated cholesterol synthesis in the liver, promote the decomposition and excretion of cholesterol from the liver, and reduced the serum level of cholesterol. Meanwhile, atorvastatin and GEFA01 downregulated the expression of *Srebp1-C, Fas*, and *Scd1*, which were related to lipid synthesis (64–66). It remains to be seen how GEFA01 influenced the expression levels of *Srebp* and *Lxr*.

Intestinal metabolites such as SCFAs play a critical role in the prevention and treatment of metabolic syndrome, bowel disorders, and certain types of cancer (67). SCFAs are crucial for intestinal health as they mediate the interaction between the diet, the gut, and the host. SCFAs are capable of regulating energy metabolism in the muscle, liver, and adipose tissues (68). The addition of SCFAs to the diet of hamsters on a high-cholesterol diet showed an upregulation of *Srebp2, Ldlr*, and *Cyp7a1* genes in the liver, thereby promoting hepatic uptake of serum cholesterol and fecal excretion of bAs (69). Meanwhile, SCFAs have been shown to inhibit HMGCR activity, thus preventing cholesterol synthesis (70), which are consistent with our results. Our results showed a significantly increased fecal acetate content in the HCD + GEFA01 group compared with the HCD group, while there were no significant differences in propionate and butyrate between the HCD-GEFA01 and HCD groups. Acetates tend to lower the concentration of serum cholesterol and have been reported to be potential agents for preventing metabolic syndrome by reducing obesity in rats and even obese human subjects (71, 72). It is reported that ~95% of the conjugated bile acids in the ileum of the intestine are reabsorbed and returned to the liver *via* the enterohepatic circulation (73), whereas the deconjugated bile acids that are not absorbed in the ileum are excreted as the feces. Total bile acid was significantly increased in feces of mice in the HCD + GEFA01 group compared to the HCD group. It is reported that the addition of SCFAs to the diet lowered the pH value in the intestine and enhanced the hydrolysis of conjugated bile acids to unconjugated bile acids (69), therefore, we inferred that increasing SCFAs downregulated the expression of *Hmgcr*, upregulated the expression of *Ldlr* and *Cyp7a1* genes in the liver, and promoted fecal excretion of bile acids, thereby indirectly reducing plasma cholesterol concentration.

Accumulating evidence has demonstrated that gut microbiota dysbiosis is closely related to metabolic diseases such as obesity, inflammatory bowel disease, and CVD (74–76). The change in the gut microbiota composition could lead to metabolic disorders by altering the production of intestinal metabolites, including SCFAs, Bas, and LPS (77). The use of probiotics such as *Lactobacillus, Bifidobacterium*, and *Enterococcus* can improve the host's gut microbiome balance to prevent the development of metabolic disorders (78, 79). In the present study, *E. faecium* GEFA01 effectively shaped the gut microbiota community. Specifically, a higher abundance of *Akkermansia* spp, *Lactobacillus*, and *Bifidobacterium* was observed in the HCD + GEFA01 group than in the HCD group. *Akkermansia* plays a role in reducing fat accumulation, and the abundance of *Akkermansia* is negatively related to total body fat content and liver triacylglycerol (80). *Akkermansia* spp, *Lactobacillus*, and *Bifidobacterium* are also reported to be well-described as SCFA-producing bacteria or belong to families with a high capacity of SCFA production (81–83). Additionally, BSH activity is possessed by several Gram-positive intestinal bacteria such as *Enterococcus, Bifdobacterium*, and *Lactobacillus* (39, 84, 85). BSH-producing probiotic bacteria have a positive effect on lowering serum cholesterol levels through deconjugation of bile salts. Enrichment of the proportion of the beneficial *Lactobacillus* and *Akkermansia* genera also correlates with increased gastrointestinal levels of unconjugated bAs including chenodexycholic acid and lithocholic acid (86, 87), which are deconjugated from conjugated bAs by BSH activity. Deconjugated bile salts are less absorbed in the intestine and excreted in the feces, and the synthesis of new bile salts from cholesterol can indirectly reduce TC concentration in the body (23), which is consistent with our results (Figure 6B). In brief, *E. faecium* GEFA01 might modulate the composition of gut microbiota, and the altered gut microbiota affected the production of metabolites such as SCFAs and bAs.

## Conclusions

**Figure 9:**
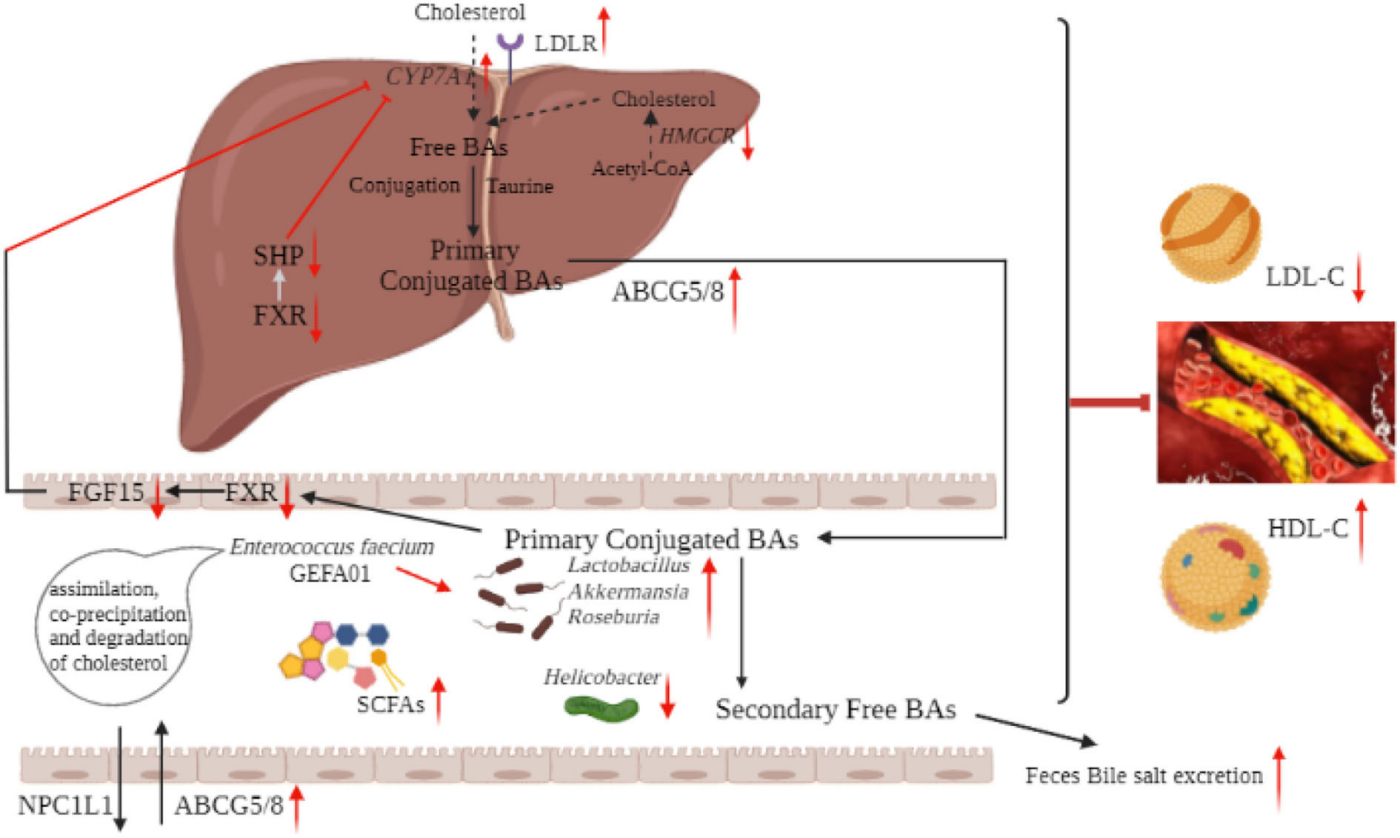
Schematic diagram of the mechanism of hypocholesterolemic effect of *E. faecium* GEFA01. Red arrows indicate an increase or decrease of expression, and light blue arrows indicate a regulation.

In summary, our results suggested that *E. faecium* GEFA01 exhibited cholesterol-lowering effects partially through assimilation, coprecipitation, and degradation of cholesterol and partially through modulation of the gut microbiota-SCFA axis. Oral administration with the strain GEFA01 increased the abundance of *Lactobacillus, Akkermansia, Bifidobacterium*, and *Roseburia* and decreased the relative abundance of *Helicobacter*. The increased abundance of *Lactobacillus, Akkermansia*, and *Roseburia* promoted the production of SCFAs. SCFAs downregulated the expression of *Hmgcr, Srebp-2, Npc1l1, Fxr*, and *Shp* and upregulated the expression of *Ldlr, Abcg5*/*8, Abca1, Cyp7a1*, and *Lxr*, thereby promoting reverse cholesterol transport and bile acid excretion (Figure 9).

## Data availability statement

The data presented in the study are deposited in the https://www.ncbi.nlm.nih.gov/, accession number PRJNA880363.

## Ethics statement

The animal study was reviewed and approved by the Nanchang University (No. 0064257) Animal Ethics Committee.

## Author contributions

LQ and HW guided and completed the whole experimental design and reviewed and revised the manuscript before submission. WX, KZ, YZ, and YC performed the experiments. LQ and WX was responsible for data arrangement and analysis. ZZ and XT provided a critical discussion of the data and revised the manuscript. WX participated in interpreting the results and wrote the initial draft with all authors providing critical feedback and edits for subsequent revisions. All authors contributed to the article and approved the submitted version.

## Funding

This research was supported by the National Natural Science Foundation of China (82160791, 81860090, 32060030, and 32101915), Natural Science Foundation of Jiangxi Province (20202BABL206007), Scientific Research Foundation of the Education Department of Jiangxi Province (GJJ190677), and PhD Research Startup Foundation of Jiangxi University of Traditional Chinese Medicine (2019WBZR009).

## Conflict of interest

The authors declare that the research was conducted in the absence of any commercial or financial relationships that could be construed as a potential conflict of interest.

## Publisher's note

All claims expressed in this article are solely those of the authors and do not necessarily represent those of their affiliated organizations, or those of the publisher, the editors and the reviewers. Any product that may be evaluated in this article, or claim that may be made by its manufacturer, is not guaranteed or endorsed by the publisher.
